# Primary ovarian CIC-rearranged sarcoma in a child: a rare case report and review of the literature

**DOI:** 10.3389/fmed.2025.1613510

**Published:** 2025-10-31

**Authors:** Liqin Ke, Lili Liu, Ying Wu, Yun Sun, Yongjiao Wang, Jiahui Xia, Wen Zhang

**Affiliations:** ^1^Department of Pathology, Wuhan Children’s Hospital (Wuhan Maternal and Child Healthcare Hospital), Tongji Medical College, Huazhong University of Science & Technology, Wuhan, Hubei, China; ^2^Imaging Center, Wuhan Children’s Hospital (Wuhan Maternal and Child Healthcare Hospital), Tongji Medical College, Huazhong University of Science & Technology, Wuhan, Hubei, China

**Keywords:** child, ovary, CIC, rearranged, sarcoma

## Abstract

**Introduction:**

CIC-rearranged sarcoma (CRS) is a rare type of high-grade undifferentiated small round cell sarcoma characterized by a range of possible CIC gene rearrangements. It develops predominantly in the deep soft tissues of the limbs and trunk in young individuals (total range, 0.5–83 years; mean, 27–37 years; median, 24.5–33 years), and less commonly in bone and viscera. The occurrence of this sarcoma in the female reproductive tract is very rare, and it has not yet been described in the ovary.

**Case presentation:**

We report a CRS case that arose from the left ovary in a 5-year-old girl. Histologically, the tumor was lobulated or leaf-shaped, with a fibrotic septum composed of closely arranged small- to medium-sized round cells and short spindle-shaped cells. In addition, the neoplastic cells exhibited multifocal membrane positivity for CD99 and diffuse positivity for WT1, TLE1, FLI‑1, P53, INI-1, and Calretinin, CD56 focal positive, while it was negative for ETV4. Fluorescent *in situ* hybridization analysis showed CIC-positive split signals.

**Conclusion:**

CRSs are highly aggressive tumors. Rare CRSs have been reported in the female genital tract, and they are often difficult to diagnose. Especially in cases with atypical morphology and immunohistochemistry, it is necessary to integrate molecular features in the diagnosis of undifferentiated neoplasms.

## Introduction

Small round cell sarcoma (SRCS) is a group of undifferentiated malignancies arising in bone and soft tissue. Ewing sarcoma (ES) is the most well-known SRCS, currently defined as having gene fusions involving one member of the FET family of genes (usually *EWSR1*) and a member of the ETS family of transcription factors (1), while undifferentiated SRCSs without FET::ETS gene fusions have been recognized and referred to as “Ewing-like sarcoma.” CIC, a human homolog of Drosophila capicua, which encodes a high mobility group box transcription factor, was found to fuse to a double homeodomain gene DUX4 as a result of a recurrent chromosomal translocation *t* (4;19) (q35; q13) in two cases of soft tissue sarcoma diagnosed as Ewing-like sarcoma (2). Recent data from pathological, molecular, and clinical studies suggest that SRCS is a heterogeneous group that includes entities other than ES. In the fifth edition of the WHO classification of soft tissue and bone tumors, the SRCS group was renamed as undifferentiated round cell sarcomas, with the incorporation of new subgroups, including the newly recognized entity of “CIC-rearranged sarcoma” (CRS) (1). Currently, there are approximately 200 cases of CRSs, with natural history, clinical behavior, and treatment outcomes reported in the literature, and less than 100 cases include clinical follow-up or treatment information. They have been reported in patients of almost any age, with a mean age of 32 years (range, 0.5–81 years) and a slight male predominance, but relatively a few cases have been documented in children (3). Most tumors occur in the soft tissue (86%), predominantly deep-seated and equally divided among trunk and extremity, followed by visceral locations (12%) and rarely in the bone (3%) (4). Rare cases have recently been reported in the female genital tracts, including vulvar, labium, cervix, and uterus (5–7). To our knowledge, CRSs in the ovary have not been reported to date.

Here, we present a case of CRS arising from the ovary in a child, the first of its kind to be reported in the literature, and discuss the clinicopathologic features, molecular genetics, diagnosis, differential diagnosis, and treatment and follow-up of the tumor, comparing them to relevant data.

### Case presentation

**Figure 1:**
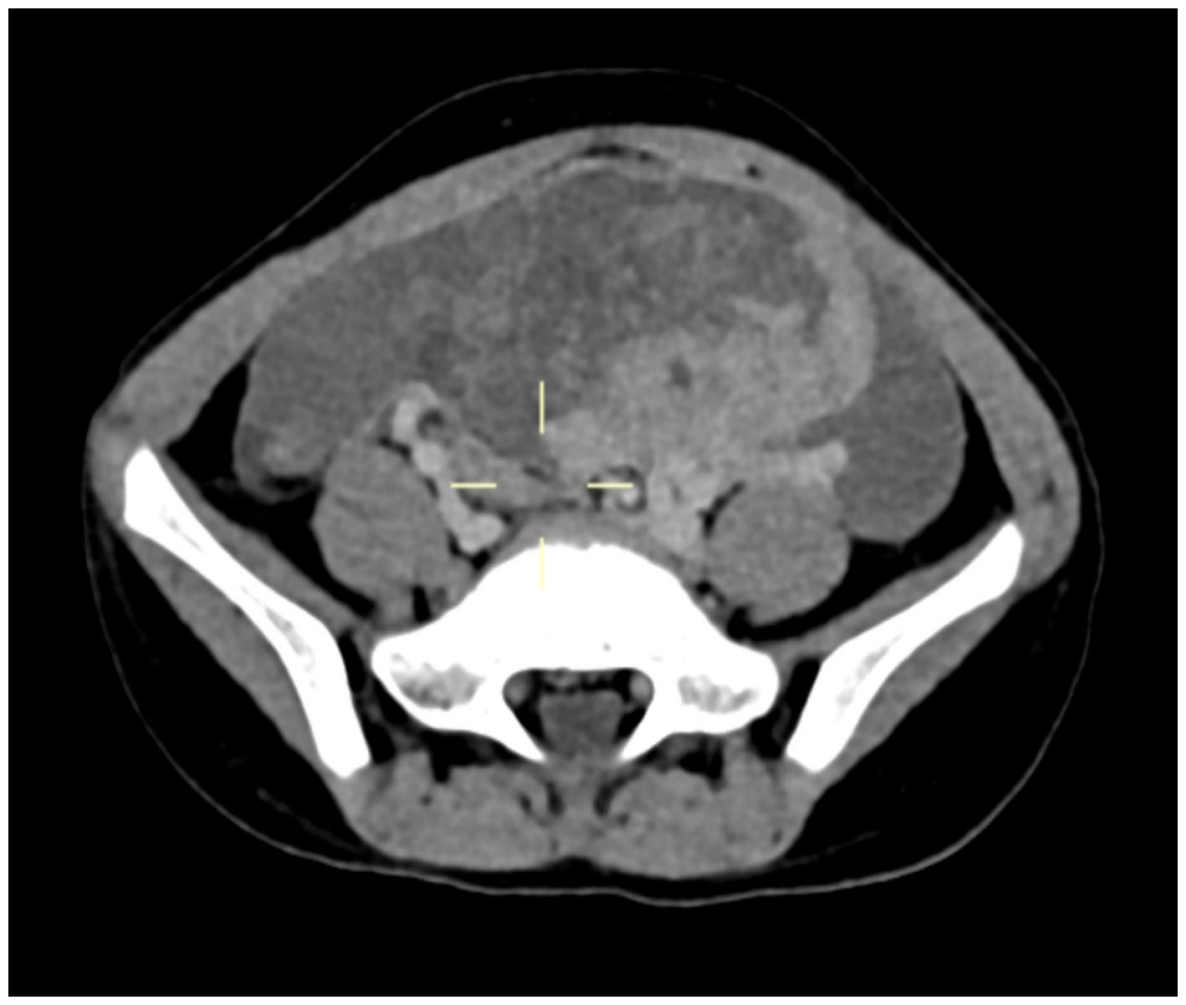
Computed tomography (CT) scan indicated a mixed-density mass shadow approximately 78 mm × 46 mm × 73 mm.

A 5-year-11-month-old girl was admitted to Wuhan Children’s Hospital (Wuhan Maternal and Child Healthcare Hospital) on 20 January 2022 due to paroxysmal periumbilical pain persisting for 4 days. On physical examination, there was positive tenderness in the lower abdomen, but there was no rebound pain or palpable mass. The levels of tumor markers CA125 and CA19-9 in serum were elevated, while serum carcinoembryonic antigen (CEA) and alpha-fetoprotein (AFP) levels were normal. The computed tomography (CT) scan showed a mixed-density mass shadow from the lower abdomen to the pelvic cavity, measuring 78 mm × 46 mm × 73 mm, with a CT value range of about 14–81 HU and unclear boundaries (Figure 1). An exploratory laparotomy was performed on 22 January 2022, which revealed peritoneal edema and tumor rupture originating from the left ovary. The bladder floor, omentum, small intestine, cecum, appendix, and anterior wall were covered with the ruptured tumor tissue and adhered tightly, making it difficult to separate. No abnormalities were found in the bilateral fallopian tubes and the contralateral ovary. A wide excision of the ovarian lesion, omentum, and peritoneum was performed along with appendectomy.

## Pathological findings

### Macro-examination

A pile of gray–brown fragmented tissue measuring 9 × 8.5 × 3.5 cm was obtained, with a light gray-brown section, brittle texture, and large areas of necrosis.

### Microscopic observation

**Figure 2:**
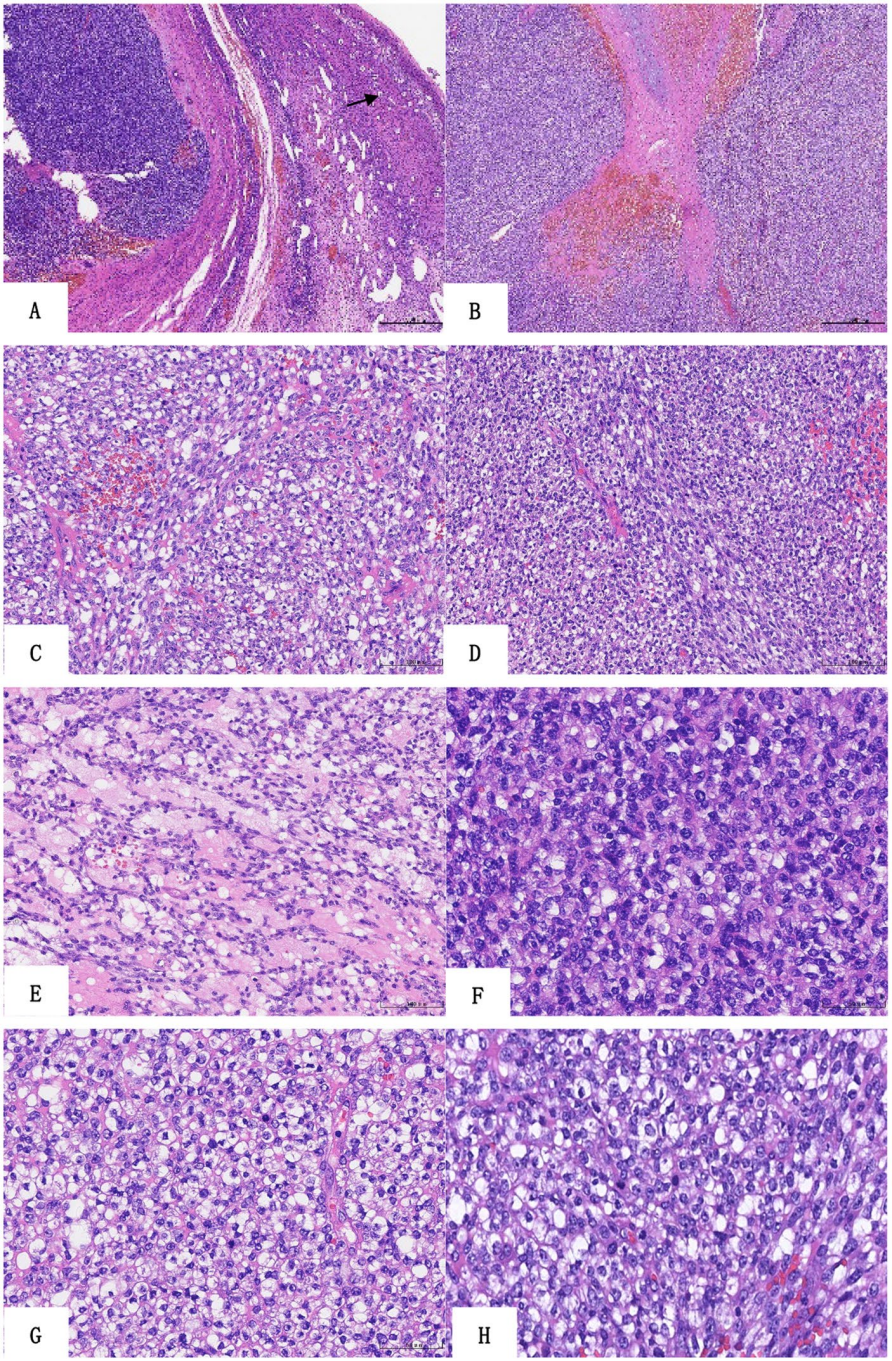
Histologic findings of the sarcoma. **(A)** The tumor shows lobulated growth in the ovary (HE ×40, follicle). **(B)** The tumor is leaf-shaped with a fibrotic septum, necrosis, and hemorrhage (HE ×40). The neoplasm grows in diffuse nests **(C)** or sheets **(D)** composed of relatively uniform small- to medium-sized round cells and short spindle-shaped cells (HE ×200). Focal reticular myxoid changes between tumor cells (**E**, HE ×200). The tumor cells have minimal nuclear pleomorphism, the nuclear chromatin is vesicular, and nucleoli are readily visible (**F**, HE ×400). The cytoplasm is little or clear cytoplasm (**G**, HE ×400), and there are scattered mitotic images between tumor cells (**H**, HE ×400).

At low magnification, the tumor was lobulated or leaf-shaped in the ovary with a fibrotic septum, necrosis, and hemorrhage (Figures 2A,B). At middle magnification, the neoplasm grew in diffuse nests or sheets comprising relatively uniform small- to medium-sized round cells and short spindle-shaped cells (Figures 2C,D), and there were focal reticular myxoid changes between tumor cells (Figure 2E). The tumor cells contained round to oval nuclei, with minor variability in size and shape (Figure 2F) at higher magnification. The nuclear chromatin was vesicular, and nucleoli were readily visible (Figure 2F). The cytoplasm was little or clear cytoplasm (Figure 2G), and scattered mitotic images between tumor cells were observed (Figure 2H).

**Figure 3:**
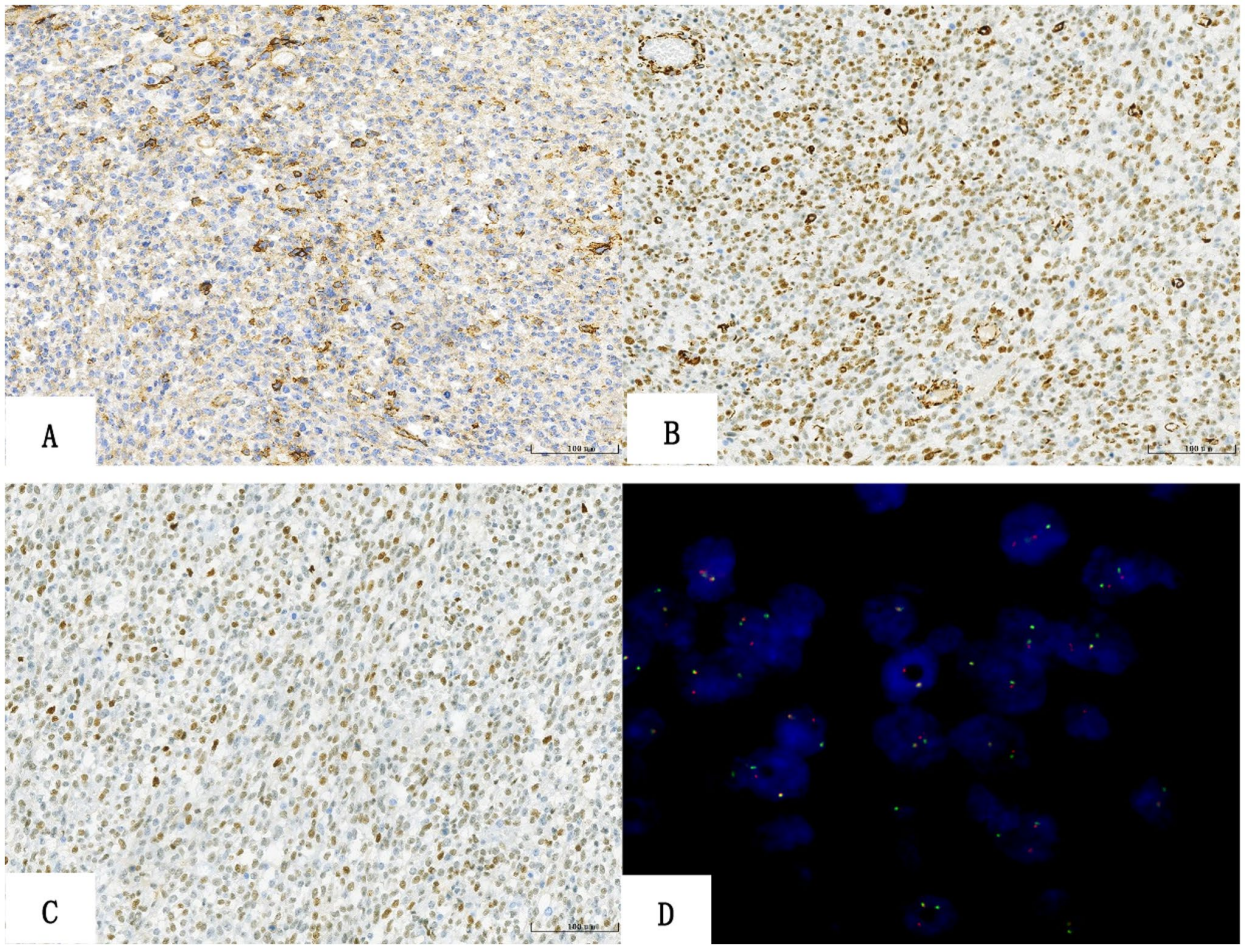
Immunohistochemical molecular features of sarcoma. **(A)** CD99 staining exhibited focal membrane positivity. **(B)** WT1 staining exhibited diffuse strong positivity. **(C)** Tumor cells showed weak positive TLE1 nuclear staining. **(D)** FISH examination revealed CIC gene rupture. (The split red and green dots in the nucleus represent gene rearrangement, while the yellow dots represent no rearrangement, accounting for = 44%).

Immunohistochemical staining provided the following results: the tumor cells exhibited multifocal membranous positivity for CD99 (Figure 3A), diffuse positivity for WT1 (Figure 3B), TLE1 (Figure 3C), FLI-1, INI-1, vimentin, and p53, and focal positivity for calretinin and CD56. The cells were negative for ETV4, cytokeratin, epithelial membrane antigen (EMA), CD34, ERG, S-100, synaptophysin, CD10, leucocyte common antigen (LCA), desmin, myogenin, MyoD1, CK7, CK20, CK5/6, HMB45, NKX2.2, BCOR, Pan-TRK, SALL-4, *α*-Inhibin, SF-1, and SMA. The periodic acid-silver methenamine (PASM) staining was also negative, and the Ki67 index was approximately 40%.

### Fluorescent *in situ* hybridization examination

Molecular analyses with FISH showed aberrant positive break-apart signals for the CIC gene (44 out of 100 evaluated tumor cell nuclei; Figure 3D).

### Pathological diagnosis

The patient was diagnosed with CIC-rearranged sarcoma (CRS) of the left ovary. The tumor components could be seen on the surface of the appendix and within the tissues of the anterior abdominal wall and the greater omentum.

### Treatment and follow-up

The patient received the vincristine, pirarubicin, and cyclophosphamide regimen (four cycles) and the ifosfamide and etoposide regimen (two cycles) in sequence as chemotherapy at our hospital, and the combination of vincristine, cyclophosphamide, and doxycycline (one cycle) at Tongji Hospital in Wuhan from January to June 2022. Five months after her initial presentation, color Doppler ultrasonography showed significant growth of the tumor. The patient continued chemotherapy at Shandong Cancer Hospital (specific details unknown). In January 2023, the patient underwent surgery and chemotherapy again after the tumor recurred, and chemotherapy ended in April 2024. Currently, oral treatment with anlotinib hydrochloride is underway.

## Discussion

CIC-rearranged sarcoma (CRS) is a group of high-grade undifferentiated small round cell sarcomas with molecular CIC gene rearrangement, categorized as a separate entity in the current WHO classification. Its morphology and immunohistochemistry findings are similar to those of Ewing sarcoma (ES), and such lesions have been diagnosed as Ewing-like sarcoma or undifferentiated round-cell sarcoma in the past. However, it shows more aggressive clinical behavior and distinct morphological and molecular features compared to ES. CRS was identified in primitive mesenchymal tumors with t (4; 19) (q35; q13.1) changes several decades ago (8). In 2006, scholars began to report that the translocations t (4; 19) (q35; q13) and t (10; 19) (q26; q13) resulted in the formation of CIC-DUX or CIC-DUX4L fusion genes (2). These genetic alterations were most frequent in *EWSR1* fusion-negative undifferentiated round cell sarcomas. CRS was found in approximately 68% of *EWSR1*-negative cases (9, 10). By far, the most common CIC gene fusion partner is DUX4, and non-DUX4 fusion partners, such as FOXO4, LEUTX, CITED1, AXL, SYK, and NUTM1 or NUTM2A, have also been reported and detected in approximately 5% of cases (11–17).

CRS occurs in all age groups but is more common in young individuals (median range, 24.5–33 years) with a slight predominance in males. Nearly 22% of cases occur in the pediatric age group (<18 years of age) (4). It mainly occurs in soft tissues in 86% of cases, and most tumors develop in the soft tissues of the trunk and limb or the head and neck region. Primary occurrence in bones is rare (4). Visceral locations account for approximately 10% of reported cases, including the lung, stomach, small intestine, colon, prostate, spermatic cord, kidney, heart, and brain. Tumors are rarely reported in the female reproductive system, with a total of no more than 10 cases with specific content reported. While several cases of CRS have been referenced in the pelvis/perineum/retroperitoneum in 2017 (4), detailed descriptions, including clinical course and treatment, have not been provided. In 2020, Ko et al. (5) reported two cases of CRS on the cutaneous of the labia/vulva in a 58-year-old woman and a 14-year-old girl. The tumors were composed of nodules/sheets of round cells with necrosis and hemorrhage separated by dense hyaline bands. Immunohistochemistry was positive for CD99 and DUX4, and fluorescence *in situ* hybridization (FISH) was positive for CIC rearrangement. Targeted next-generation sequencing (NGS) was positive for CIC-DUX4 fusion. In the same year, Sedighim et al. (6) reported on a 28-year-old female with undifferentiated round cell sarcoma with CIC rearrangement in the uterus. In 2023, Zhao et al. (7) reported two cases with diffuse patchy undifferentiated round cells in a nodular pattern, which occurred in the uterine fundus and cervix of postmenopausal women, with CIC rearrangement. In this study, we present and discussed a case of CRS in the ovary, the first of its kind to be reported in the literature. According to the data in Table 1, CRS of the female genital tract is a rare subset that most commonly involves the uterus. It is different from ES, which usually occurs on the ovaries (18).

**Table 1:** Summary of CIC-rearranged tumors in the female genital tract.

Case	Age (year)	Site	FISH CIC	Targeted NGS	Recurrence/metastasize	Follow-up/death (mos)	References
1	14	Vulvar	+	ND	N/Y-lungs	23	Ko et al. (5)
2	58	Labium	ND	CIC-DUX4	N/N	12	Ko et al. (5)
3	28	Uterus	+	ND	Y/Y-liver	14/death	Sedighim et al. (6)
4	58	Uterine fundus	+	ND	Unknown	Unknown	Zhao et al. (7)
5	59	Cervix	+	ND	Unknown	Unknown	Zhao et al. (7)
6	5	Ovary	+	ND	Y/N	40	Our case

FISH, fluorescence *in situ* hybridization; NGS, next-generation sequencing; ND, not detected.

CRS shows a more heterogeneous appearance when compared with ES (19). It is composed of small- to medium-sized round or oval cells arranged in a nodular, lobulated, or flake-like pattern separated by fibrous connective tissue. Geographic necrosis and hemorrhage are common findings, and the mitotic count is usually high. The tumor cells have scant cytoplasm and prominent nucleoli. Spindle cells and epithelioid cells may be seen in many cases. In rare cases, neoplastic cells adopt an epithelioid morphology, with occasional rhabdoid-like cytoplasm or a clear cell change in the cytoplasm (20, 21). Stromal myxoid change is a common finding, which is typically absent in ES (22). In our case, the tumor was lobulated, composed of small- to medium-sized round cells and short spindle-shaped cells, with geographical necrosis and hemorrhage. The tumor cells were relatively uniform, but the nuclei showed minimal pleomorphism, with little or no clear cytoplasm and prominent nucleoli. In addition, myxoid changes were observed between tumor cells. Therefore, the growth pattern and cell morphology of the tumor were consistent with those reported in previous studies.

CRS exhibits a “scrambled” immunophenotype, which may lead to diagnostic challenges. The staining intensity of CD99 varies, from focal weak positive to negative. Past studies suggest that CD99 was focal positive in ~85% of cases, but only 23% reported a diffuse pattern and 16% were completely negative, which was different from the strong diffuse positivity in ES (4). ETV4 is a transcriptional target of CIC-DUX4, and the specific oncogenic translocation CIC::DUX4 can induce ETV4 overexpression. Diffuse nuclear positivity with 70–95% WT1 expression has suggestive value in the diagnosis of CRS (4, 22). According to Hung et al. (23), the sensitivity and specificity of diffuse ETV4 expression for CRS were 90 and 95%, respectively, whereas the sensitivity and specificity of WT1 were 95 and 81%, respectively. The expression of WT1 and ETV4 is consistently present, which is useful for the differential diagnosis of CRS, although not entirely specific. In the report by Siegele et al. (24), DUX4 was diffuse nuclear positive in CIC-DUX4 sarcoma, with a sensitivity and specificity of 100%. The CRS was also reported to have the following features: ERG, TLE1, and CD56 positivity and occasional desmin, S-100, MUC4, epithelial membrane antigen (EMA), AE1/AE3, and calretinin focal positivity (22). Our case exhibited multifocal CD99 expression and diffuse positivity for WT1, TLE1, FLI-1, P53, INI-1, and calretinin, with focal positivity for CD56. All of these features were basically consistent with those reported in previous studies. However, unlike what Hung et al. (23) reported, our current pathology does not stain positively for ETV4, which may be related to the CIC gene fusion partner. Therefore, the definitive diagnosis of CRS depends on the cytogenetic abnormalities of the tumor.

A CRS occurring in the ovary should be differentiated firstly from the following diseases: (i) Granulosa cell tumor—the tumor is composed of round and oval cells diffusely arranged in a sheet-like structure, with nuclear grooves and unique Call–Exner bodies. In addition, it may be distinguished by immunostaining expression of *α*-inhibitors, calretinin, FOXL2, SF-1, WT1, and CD56. (ii) Hypercalcemia small cell carcinoma—the tumor generally grows diffusely with small and uniform cells, deeply stained nuclei, coarse chromatin in clusters, small nucleoli, and many mitotic figures. The patient often suffers from hypercalcemia in this type of tumor. Tumor cells are generally diffusely positive for WT-1 and often locally positive for cytokeratin (CK), EMA, CD10, and calretinin. However, CIC rearrangement is negative. (iii) Endometrial stromal sarcoma of the ovary—the small and uniform tumor cells grow in sheets, with scant cytoplasm and bland oval nuclei with inconspicuous nucleoli. These tumors typically express CD10, estrogen receptors (ERs), and progesterone receptors (PRs), with variable expression of CK and nuclear WT-1. (iv) Poorly differentiated neuroendocrine carcinoma—poorly differentiated epithelial cells have a nest-like structure but are positive for epithelial/neuroendocrine markers and do not exhibit CIC rearrangements.

CRS mostly needs to be differentiated from ES and other Ewing-like undifferentiated small round cell sarcomas, such as *BCOR*-rearranged sarcoma. However, both are small round cell tumors and have scant cytoplasm. CRS shows significantly higher degrees of lobulation, nuclear pleomorphism, the prominence of the nucleoli, spindle cell elements, and myxoid changes. Immunohistochemically, CRS also has several characteristics distinct from ES and Ewing-like sarcomas. CD99 usually shows weak to moderate positivity and partial staining, thereby lacking the strong and diffused membrane pattern observed in ES. Other distinguishing immunohistochemical features include strong WT1, TLE1, ETV4, and DUX4 expression in CRS. In contrast, NKX2.2 is expressed in ES, and *BCOR* is expressed in *BCOR*-rearranged sarcoma, and it can be differentiated from CRS based on EWSR1-FLI1, *EWSR1-ERG*, *BCOR*, and the expression of other related genes.

Although immunohistochemistry may help with the diagnosis of CRS, the identification of molecular features is necessary. In clinical settings, FISH dual-color break-apart probes have been widely used to detect the rearrangement of the CIC gene. It should be noted that CIC break-apart FISH has also been shown to miss a significant subset of CIC-DUX4 sarcomas. Yoshida et al. (25) demonstrated that the detection of FISH probe had a false–negative rate of 14%; therefore, a negative result cannot completely rule out CRS, and RT-PCR, tri-color FISH detection, or second-generation sequencing can be employed if required.

Currently, patients with CRS are prescribed the same therapeutic protocol as ES, with an anthracycline-based regimen, surgery, and radiotherapy (26). Approximately 40% of CRS patients show evidence of metastatic disease at initial diagnosis, mainly involving the lung (4, 22, 27), and 70% of CRS cases respond poorly to chemotherapy. The 5-year overall survival rate is 17 to 43% (22, 27, 28), which is significantly lower than the 80% 5-year overall survival of ES patients (4, 22, 27). The median survival rate is 12 to 18 months (22, 27, 28), and the prognosis of these patients is significantly worse than that of patients with ES. Our case reported recurrence 5 months after undergoing surgery and multiple chemotherapy treatments and relapsed again 1 year later. Currently, the patient is on oral Anlotinib for maintenance therapy.

In conclusion, we have reported a rare case of CRS arising from the ovary and discussed the clinicopathological features, diagnosis and differential diagnosis, and treatment and prognosis of the tumor. Combining relevant literature, CRS of the female reproductive tract is very rare and can occur at any age (Table 1). Its histomorphological and immunophenotypic features are similar to those reported at deeper anatomical locations. In case of molecular analyses with FISH, all cases show CIC rearrangement, with only one case indicating fusion of CIC-DUX4 on next-generation sequencing (NGS) analysis. The other pairing genes for the CIC gene fusion have not been discovered yet. It is difficult to diagnose CRS arising in the female reproductive tract and requires detailed analysis of morphologic features, immunohistochemical findings, and molecular genetics. CRS progresses rapidly and has a high metastasis rate. At present, patients are on a treatment methodology similar to ES and exhibit a worse prognosis than ES. To date, a specific and effective treatment for CRS has not been defined.

## Data Availability

The original contributions presented in the study are included in the article/supplementary material, further inquiries can be directed to the corresponding author.
